# How do animal territories form and change? Lessons from 20 years of mechanistic modelling

**DOI:** 10.1098/rspb.2014.0231

**Published:** 2014-06-07

**Authors:** Jonathan R. Potts, Mark A. Lewis

**Affiliations:** 1 Department of Mathematical and Statistical Sciences, Centre for Mathematical Biology, University of Alberta, Edmonton, Alberta, Canada T6G 2G1; 2 Department of Biological Sciences, University of Alberta, Edmonton, Alberta, Canada T6G 2G1

**Keywords:** animal movement, home range, individual-based models, mechanistic models, reaction–diffusion equations, territoriality

## Abstract

Territory formation is ubiquitous throughout the animal kingdom. At the individual level, various behaviours attempt to exclude conspecifics from regions of space. At the population level, animals often segregate into distinct territorial areas. Consequently, it should be possible to derive territorial patterns from the underlying behavioural processes of animal movements and interactions. Such derivations are an important element in the development of an ecological theory that can predict the effects of changing conditions on territorial populations. Here, we review the approaches developed over the past 20 years or so, which go under the umbrella of ‘mechanistic territorial models’. We detail the two main strands to this research: partial differential equations and individual-based approaches, showing what each has offered to our understanding of territoriality and how they can be unified. We explain how they are related to other approaches to studying territories and home ranges, and point towards possible future directions.

## 1. Introduction

Territoriality occurs widely throughout the animal kingdom, observed in taxa as diverse as mammals, birds, insects and fishes. Territories are spatial regions, defended against conspecifics, for the purpose of using resources and providing mating opportunities. Different species use a wide variety of tactics to defend territories, such as deposition of visual or olfactory cues, fighting or ritualistic displays, with records of such behaviour dating back as far as the seventeenth century [1,2]. While theoretical biology has a rich history of analysing pattern generation from microscale interactions to macroscale [3], it was not until about 20 years ago that population-level territorial patterns were modelled analytically as emerging from individual-level interaction events between similar animals [4] (although see [5] for an early example of segregation emergence between animals with highly differing behavioural traits).

Historically, much modelling of space use has been based on phenomenological descriptions of the areas used by animals, such as drawing a minimum convex polygon around location fixes to construct a plausible home range [6], or assuming that space use will correspond with food availability, as with resource selection analysis [7]. These approaches have become increasingly sophisticated over the years, through models such as kernel density estimators and Brownian bridges, leading to realistic descriptions of spatial patterns [8,9].

While accurate description is valuable, prediction requires a solid understanding of the individual-level mechanisms that give rise to observed spatial patterns. The construction of quantitative, predictive ecology that can foresee the impact of environmental change on species' ability to survive and reproduce is a vital challenge for twenty-first century science. The rapid changes currently occurring in many of the Earth's ecosystems force animals to respond before they can adapt, attempting as best they can to make use of the new environments they find themselves in. By uncovering the behavioural mechanisms underlying their choice of area as they move and interact with one another, it may become possible to predict the effects of habitat variation on the spatial structure of a population.

On the more theoretical side, mechanistic territorial models provide a key step towards constructing a statistical mechanics for ecological systems [10]. This programme seeks to find quantitative theories explaining how ‘macroscopic’ ecosystem patterns derive from ‘microscopic’ individual processes, in analogy with the laws linking macroscopic physical properties, such as pressure and temperature, to the behaviour of the underlying system of molecules. Although ecosystems containing living creatures are more complex than collections of molecules, the general principle of beginning with a random walk model, then using mathematical analysis to derive properties of the system, has already borne much fruit in movement ecology research [11,12]. Therefore, scientists are gradually moving towards the goal of building a predictive ecological theory based on the concept of statistical mechanics, by constructing the jigsaw puzzle one piece at a time.

The specific puzzle-piece that relates to building models of space use from territorial *interactions*, which is the focus of this review, began when Lewis & Murray [4] constructed and analysed one-dimensional advection–diffusion equations based on scent deposition of wolves and the subsequent avoidance response of neighbouring packs. Since then, this formulation has been refined to take account of the precise details of movement and interaction events, generalized to the biologically realistic two-dimensional case and extended to account for environmental effects [13,14]. It has been successfully used to test hypotheses about the underlying causes of territory size and shape and demonstrate the effects of population change on territorial structure [15]. Recently, it has also been applied in the sociological context of human gang territories [16]. This wide variety of applications both demonstrates that there exist general mathematical structures behind the multifarious territorial interaction mechanisms residing in the natural world, and shows the effectiveness of mechanistic modelling at answering hitherto unsolved biological questions.

Three years ago, there was a further fundamental advancement in our understanding of territory formation and dynamics [17]. The authors stripped down the model of scent-mediated conspecific avoidance to a very simple, individual-based model (IBM) of territory formation, which was analysed from the ground up, without taking the mean-field limits used in the advection–diffusion approach [15]. Although qualitatively similar territory patterns emerged in this model, it also displayed a number of features not present in the advection–diffusion approach, most notably details of the timescale over which territory boundaries shift, as well as an ability to quantify the longevity of scent mark cues purely by examining the evolution of animal locations over time [18,19].

The purpose of this paper is to give a detailed review of the progress in both approaches to territorial modelling, which relate mainly to terrestrial mammalian carnivores, but have recently been extended to birds [20]. We explain how a recently proposed formalism can unify the two frameworks and relate them to the fields of resource selection analysis and collective animal behaviour, two areas of ecology that have separately evolved rich histories of modelling and data analysis. Finally, we give a perspective into the future of mechanistic territory modelling and how we see its place in helping to answer pressing questions in current ecological research.

Throughout, we are careful to distinguish territorial formation from the related concept of home range emergence [21,22]. While territorial interactions are often key in the formation of home ranges, they are not a necessary mechanism, with home ranges often forming in the absence of conspecific avoidance. The underlying localization processes in these cases may, for example, result from resource attraction or site fidelity owing to memory [23]. Although the examination of home range formation in the absence of conspecific avoidance interactions is beyond the scope of this review, we include a section (‘Home range emergence’) where we explain how mechanistic territory models fit into the general effort of understanding home range formation. For a good recent review of home range analysis, instead see [22], or [24] in the context of mechanistic modelling.

## 2. The dynamical systems set-up

Mathematical models for animal movement take a variety of forms. Normally, one might think of tracking an animal's location at fixed time intervals. In this case, a movement kernel would describe the distribution of step lengths and movement directions from one time step to the next [11,12]. However, local environmental conditions, such as terrain or prey, or territorial signals, such as scent marks, would also feed into the movement kernel [15,25]. An animal may bias movement away from steep terrain or might take shorter steps in regions with high prey density. Territorial signals, such as scent marks, could also affect the direction of movement through avoidance of foreign scent marks or attraction towards familiar scent marks [15].

To complicate matters, some of these factors, such as terrain, are external to the animals, whereas others, such as prey density or scent mark density, involve negative or positive feedbacks as animals modify their local environments, a process sometimes referred to as stigmergy [26]. One way of accounting for these effects is to create an IBM with reasonable behavioural rules that involve changes to the local environment and responses to environmental conditions. The IBM could be simulated to explore the suite of possible outcomes. A particular example of this approach is given later, in the section ‘An individual-based approach’. First, however, we examine an alternative approach, which attempts to approximate the probability density function (PDF) of the animal or group of animals.

## 3. Probability density function approach

Although the precise location of the animal at any point in time is uncertain, the range of possible locations of the animal can still be described using a PDF. This modelling approach simulates the time evolution of the PDF as territorial interactions reshape it [15]. Thus, rather than requiring multiple stochastic simulations, the PDF approach involves a single simulation that tracks the expected space use of the animal over time.

The basic tool for moving from individual descriptions to PDFs is the master equation [11,12]. This is an iterative equation that describes the PDF of the animal at a time *t* + *τ* in terms of the PDF at time *t* and a dispersal kernel describing the individual movement patterns. The kernel is based on an individual-level description of the animal's movement between *t* and *t* + *τ*. Many individual-level processes have been proposed for movement between successive locations, such as step selection functions [27], Brownian motion [9] and state–space models [28]. The master equation provides the bridge between these individual-level descriptions of movement and more ‘macroscopic’ space-use patterns described by the PDF.

The classical method for analysing master equations involves using the Fokker–Planck equation to approximate the movement model. This allows one to go from the kernel-based description of movement for individuals, including environmental conditions and feedbacks, via the master equation, to a system of advection–diffusion equations that track the expected space use over time. The resulting system of equations can be simulated on a computer or analysed mathematically to predict the emergence of territorial patterns.

Early applications of the Fokker–Planck approach focused on determining the behavioural ingredients needed for territorial pattern formation [4]. They asked: what behavioural interaction terms, including scent-marking, will give rise to the spontaneous formation of territories? The simplest model involved two packs interacting in one spatial dimension, each producing scent marks that cause avoidance movement by the other pack. Each pack was modelled as moving back towards its den site when it encountered foreign scent marks. It turns out that this simple model is sufficient to generate territories [4]. The addition of positive feedback, through enhanced scent-marking over foreign scent marks, gives rise to *bowl-shaped* patterns of scent marks, with the edges of the bowl describing heightened scent densities found at the edge of the territories [29]. It also gives rise to *buffer zones* between territories, where neither pack would go. Both these features have been studied extensively in wolf (*Canis lupus*) territories in northeastern Minnesota, and the fact that simple behavioural rules give rise to such realistic emergent patterns is a persuasive argument as to the model's validity [4].

**Figure 1.**
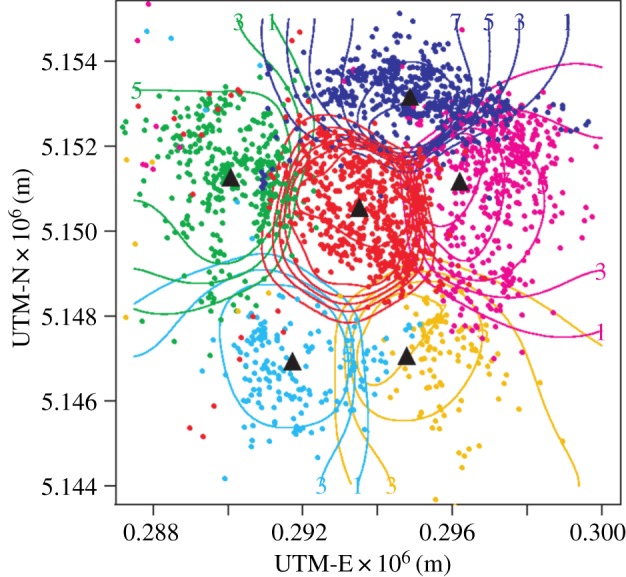
Mechanistic territorial model applied to coyote populations. These relocation data for coyote from different packs, denoted by different colours, are fitted using the method of maximum-likelihood. The model posits that animals move randomly and avoid foreign sent marks by moving back towards their den site or organizing centre (triangles). The scent marks (not shown) have their own dynamics where there is a constant low level of marking, with foreign scent marks causing an over-marking response. Full details of the model are given in Moorcroft *et al*. [13]. Reproduced with permission from Moorcroft *et al*. [13]. (Online version in colour.)

Realistic models for animal territories must include multiple spatial dimensions, as well as the spatial distribution of external factors, such as resource and topography. A second generation of sophisticated two-dimensional advection–diffusion models has been developed so as to include these factors [15]. By using the method of maximum-likelihood to connect the models with data, hypotheses about the factors driving territorial pattern formation can be tested from the space-use patterns as measured by radiotelemetry data. This method was applied to test the role of scent-marking on coyote (*Canis latrans*) territorial patterns in the Hanford Arid Lands Ecosystem [13] (figure 1) and additional impacts of topography and prey distribution on these patterns in the Lamar Valley region of Yellowstone [14]. Here, the connection between advection–diffusion models for territorial patterns and classical hypothesis testing is new, and it provides a powerful approach for connecting mechanistic movement models with data.

As these advection–diffusion models have become more mainstream, new applications have extended the modelling theory. For example, the process of shifting territories, as new groups form and old groups split, has been very recently explored using the advection–diffusion approach applied to an extensive dataset for territorial meerkats in South Africa [30]. These mechanistic models have also been reapplied in a new context, to the formation of gang territories in the Hollenbeck region of Los Angeles [16]. Here, natural barriers to gang movement, including rivers and freeways, replaced the topography component in the models.

## 4. An individual-based approach

### (a) The modelling framework

An alternative approach to advection–diffusion modelling was proposed in reference [17], whereby the animals were modelled on a discrete lattice, and analysis was performed without first taking a mean-field limit. It is well known that when interactions are rare, as is often the case with territorial animals, continuum models can give very different results to the underlying IBM [31]. Therefore, it is important to examine whether there are aspects of territoriality that exist in an individual-based approach, but are not present in reaction–advection–diffusion systems.

**Figure 2.**
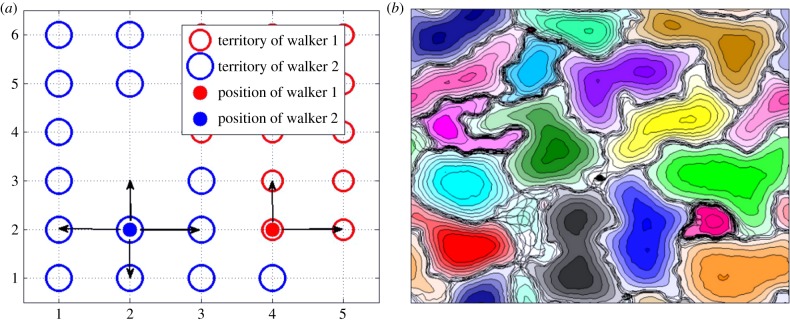
The individual-based territoriality model with example output. The left-hand panel represents a hypothetical snapshot in time of the position of two territorial random walkers (animals), the red and blue dots, and their territories, represented by the red and blue open circles, respectively. If a red (blue) open circle is present at a lattice site, it means that the red (blue) animal has been in that location sometime in the past *T*_AS_ timesteps. The absence of any scent marks at coordinates (5,1), (2,3) and (2,4) implies that no animal has occupied those coordinates within a time *T*_AS_, i.e. this is interstitial area. The next time the blue animal moves, it can go to any of the four adjacent lattice sites with equal probability, whereas the red animal is constrained to move either up or right. The right-hand panel demonstrates the sort of home range patterns that can arise from such a model. Reproduced from Giuggioli *et al*. [17]. (Online version in colour.)

The so-called ‘territorial random walk’ models animals as nearest-neighbour lattice random walkers, each of whom deposits scent as it moves, which lasts for a finite amount of time, the ‘active scent time’ (*T*_AS_), after which other (conspecific) animals no longer respond to the mark as fresh (figure 2). They are able to move to any nearest-neighbour lattice site unless the site contains active scent of a conspecific, in other words unless that site is in the conspecific's *territory* [19].

An advantage of this approach is that it provides a natural definition of the animal's territory at any point in time: the set of lattice sites containing active scent of that animal. This readily corresponds to the definition from Burt [21] of a territory being ‘any defended area’. This definition is contrasted with that of a home range, the latter being ‘that area traversed by the individual in its normal activities of food gathering, mating and caring for young’ [21]. In more mathematical terminology, this might be called the *utilization distribution* of an animal as measured over a period of time spent engaging in such ‘normal’ daily activities.

The territories that emerge from these lattice models are not static, but change slowly over time, typically much slower than the movement of the animals themselves. As a consequence, when measured over a finite time window, the utilization distributions (home ranges) of animals in adjacent territories will overlap slightly. Such overlapping home ranges are common in territorial systems, but contrast with the concept of contiguous territories or territories separated by buffer zones [4]. That both home ranges and territories emerge in conceptually separate, but clearly defined ways from this model enables rigorous qualification of the traditional descriptive differences [21].

If the movement of the animals has no intrinsic localization process, then home range overlap will steadily enlarge as the time window is increased, without ever stabilizing. The urban foxes (*Vulpes vulpes*) studied by Potts *et al*. [19] lack such a central place attraction, but many animals do have a bias in their movement towards a den or nest site [15]. Incorporating this bias into the IBM approach causes stable home ranges to emerge, despite the territory borders remaining in constant flux [18].

The main bulk of work on individual-based territorial models has so far been based on full territorial exclusion, where animals completely avoid areas containing conspecific territory marks. However, it is typical for animals to exhibit a certain amount of curiosity and probing on the territory border, pushing into recently marked areas a small amount before subsequently retreating. Indeed, such a process has recently been shown to occur in populations of Amazonian birds [20]. In reference [26], the patterns emerging from a process of partial exclusion in an IBM were studied, giving qualitatively realistic patterns of overlapping home ranges.

### (b) Mathematical analysis

An advantage of the individual-based approach is that it explains the phenomenon of moving territory borders, sometimes called the ‘elastic disc hypothesis’, which has been observed in species from a variety of taxa (see references in Potts *et al*. [19]), ever since the seminal paper of Huxley [32]. A disadvantage is that it is highly computationally intensive to fit stochastic IBMs to data.

To circumvent this issue, approximate analytic versions of the simulation models that describe the movement of animals in side fluctuating territory borders were constructed in one-dimension [33] and two-dimensions [18,34]. These were solved exactly, giving expressions that are readily fitted to data on animal movement [19]. The models are based on the observation from simulation output that territory borders exhibit slow random movement that constrain the animals' intrinsic diffusive motion. As such, parametrizing them requires knowledge of the territory border movement and they do not, in themselves, contain information about the scent-marking process. Therefore, fitting data to these models does not give any information about the active scent time.

**Figure 3.**
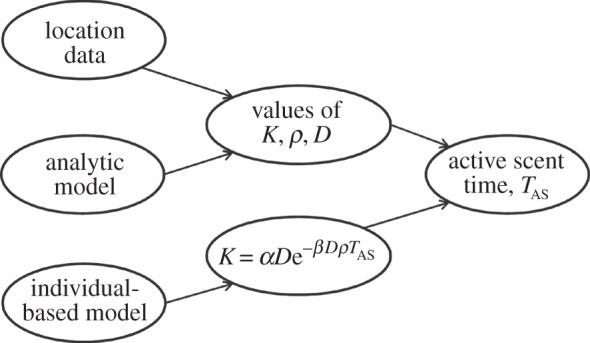
Using individual-based territoriality models to extract scent longevity from location data. Location data can be fitted to the analytic model of [34] using the methods of [19] to give information about the territory border movement, *K*, the animal's intrinsic diffusion constant, *D*, and the population density *ρ*. Analysis of the IBM from [17] then gives a universal curve *K* = α*D*exp(*−*β*D*ρ*T*_AS_) [19], which can be used, together with the information on *K*, *D* and *ρ*, to obtain an estimate of the active scent time, *T*_AS_.

However, there turns out to be a ‘parameter collapse’ of the simulation output to a universal curve relating the generalized diffusion constant of the territory border, *K*, to a dimensionless input parameter *Z*, so that 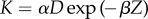 for particular constants *α* and *β* reported in reference [33] in one-dimension and [19] in two-dimensions. Here, 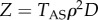 in one-dimension and 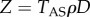 in two-dimensions, where *ρ* is the population density and *D* is the intrinsic diffusion constant of the animal. This enables users of this modelling approach to extract the active scent time from details of the border movement that, in turn, can be extracted from movement data via the approximate analytic model (figure 3).

An important, unsolved issue from this approach is to understand analytically why the parameter collapse to 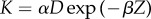 is observed, and whether it holds for all parameter values or just those analysed in references [19,33]. Some initial steps towards this end were made in reference [35], where the authors noted that this trend is related to the drift probability of one territory boundary into its neighbour, via a first passage time argument. This drift probability can be thought of as the amount of pressure one territory exerts on a neighbour. Although this surprisingly challenging mathematical problem provided a key step forward, much more needs to be done to understand fully this parameter collapse.

### (c) Ecological and epidemiological lessons

Applying this model to animal location data enables quantification of both the interaction process, that is the active scent time, and the amount of intrinsic flexibility in the territorial structure, that is the border diffusion constant *K*. By using data before and after an outbreak of mange in Bristol's red fox population [19], it was possible to quantify how both the territorial structure and the behaviour of foxes changed as the disease spread through the population.

These changes turned out to be quite dramatic, having important consequences for modelling epizootics in territorial populations. The study showed that it is not accurate to assume that the animals, even those that do not have the disease, will necessarily maintain their behavioural patterns. Large amounts of government money rely on good understanding of such disease spread, notably the recent decision to cull badgers by the UK government to stop the spread of bovine tuberculosis [36]. This decision itself was based upon the controversial notion that badgers will not change their territorial structures as a result of disturbing the population through culling [37, §§3.6.9–10]. The approach of [19] gives perhaps the first mechanistic theory that explains why such assumptions are likely to be false, so the underlying modelling framework could prove useful in helping governments make better-informed decisions.

## 5. Fit to the movement process or the territorial pattern?

When applying mechanistic territory models to data, researchers have generally tended to fit the emergent territorial patterns to relocation data, regardless of whether they have used advection–diffusion or IBM approaches [14,19]. A different approach fits models to the fine-scale movement and interaction processes, then uses them to derive the resulting space-use patterns [12]. An advantage of the former approach is that it does not rely on the availability of detailed movement data. A disadvantage is that the fitting procedure, typically based on a maximum-likelihood approach [14], requires that animal locations be independent samples of the utilization distribution. Obtaining sets of points that are approximately independent usually requires using a small subsample of the data, which can mean discarding a lot of information [15].

**Figure 4.**
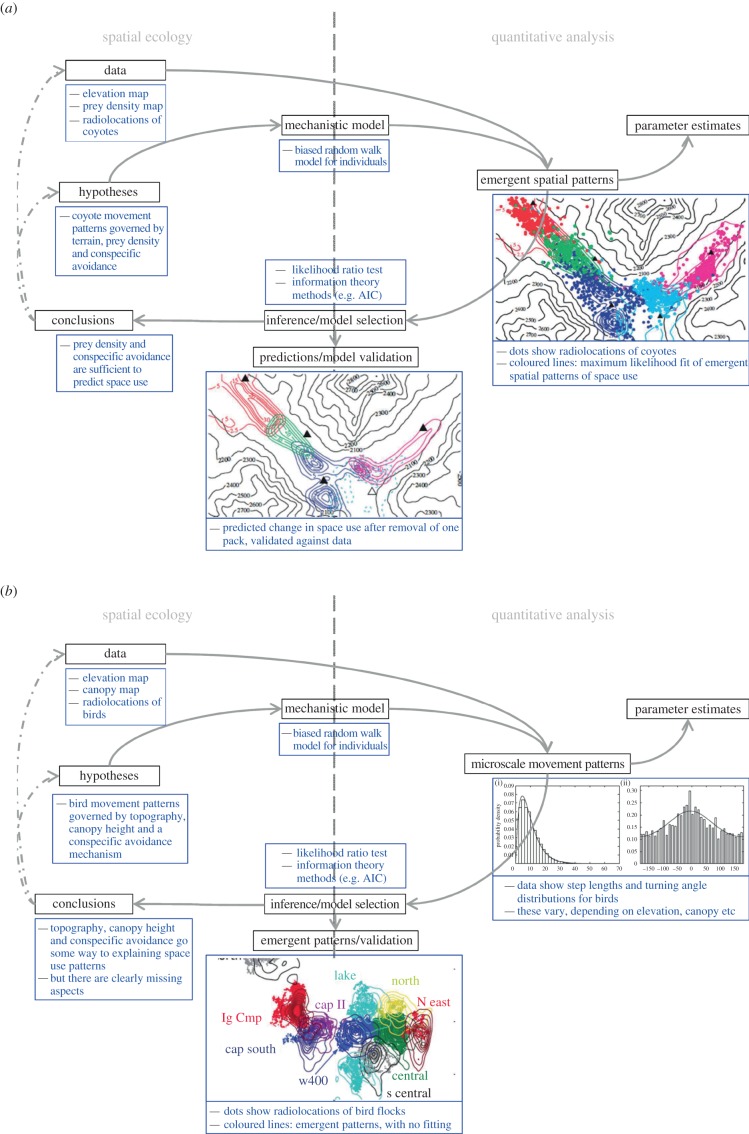
Fit to the process or the pattern? (*a*) gives a schematic of the modelling scheme for mechanistic models where we fit data to the territorial pattern [15,19]. (*b*) How this process differs when we fit the data to the movement and interaction processes [20]. (Online version in colour.)

Fitting a model directly to the underlying movement and interaction processes, on the other hand, allows one to make use of all the location data available. Owing to advances in global positioning satellite technology over recent years, fine-scaled animal movement data are becoming increasingly common, making such model fitting possible. Once such a model has been parametrized, it is possible to use either simulation or mathematical analysis to derive the resulting territorial patterns [20]. Because these patterns are not themselves fitted to the positional data, as in previous approaches, this approach is far more conservative in answering whether a model is sufficient to produce territorial patterns (figure 4).

The procedure used in this analysis is based around the notion of a *step selection function* [27], which gives the probability 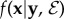 of moving from position **y** to **x**, given information about the surrounding landscape 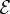. Moorcroft & Barnett [38] noted that this is precisely equivalent to the movement kernel of mechanistic territory and home range models. Therefore, by fitting step selection functions to data, using methods such as in reference [39], it is possible to parametrize a mechanistic movement model, which can, in turn, be used to derive space-use patterns in a mathematical and non-speculative fashion, using techniques developed in reference [15]. By coupling together step selection functions for different animals [20], interactions can also be explicitly incorporated in this modelling approach.

## 6. Optimality and game theory

Although a mechanistic model with fixed parameters, may, on average, describe animal movement behaviours, individuals may modulate their behavioural responses, responding to local conditions so as to optimize fitness [40]. Mathematically, this could be achieved by a modification of parameters in the mechanistic model. However, when more than one group is simultaneously involved in optimizing, the appropriate framework to describe interactions is actually in terms of a game [41].

It turns out the issue of buffer zones between wolf territories provides a fascinating context for the application of game theory. This is because there is a strong positive correlation between the locations of the buffer zones and heightened densities of the primary prey species for wolves in northeastern Minnesota, white-tailed deer. The deer appear to thrive in these buffer zones owing to reduced predation pressure. This begs the question as to why the territorial wolves do not simply trespass into the buffer zones between territories and consume the precious prey species before neighbouring packs take the opportunity. After all, animals are seldom mindless automata, obeying fixed behavioural rules, and it is natural to ask how these rules might adapt so as to maximize fitness.

The idea that territorial movement behaviour can be modified so as to improve a wolf pack's fitness is quite reasonable biologically but is a challenge to address quantitatively [41]. An early attempt to model optimal behavioural responses of territorial wolves in this complex spatial predator–prey dynamic used the theory of differential games to show conditions under which buffer zones would persist and why they might break down [42]. Packs were assumed to modulate their movement behaviour so as to attempt to maximize food intake while minimizing the chance of hostile altercations with neighbours. A key result from this analysis showed that buffer zones can persist as evolutionarily stable outcomes, providing the penalty for interpack altercation is high, and, crucially, providing there always remains a random component of movement, describing the uncertainty inherent to wolf movement. This area of coupling spatially explicit territorial models to game theory is in its infancy, and there is a real opportunity for new analysis.

## 7. The related concept of home range

Any animal that maintains a territory will ipso facto have a home range. However, the converse is not true. Many animals exhibit home range behaviour without actively defending a territory, for example caribou herds [43]. Consequently, much effort has gone into examining the mechanisms that cause the formation of home ranges in the absence of conspecific avoidance processes (e.g. see Grimm & Railsback [44] for various IBM approaches to this). Although we focus here on models that incorporate territorial interactions, it is worth giving a brief overview of other home range models as they are often closely related. Detailed reviews can be found elsewhere [22,24].

Models of home range emergence in the absence of territorial interactions typically involve fidelity to a particular place or places. To generate this fidelity, models often assume that there is an underlying memory process [45]. As animals move, they will remember where they have gone in the recent past and modify their future movements accordingly. These modifications may cause biases towards sites that they have recently visited [23], towards patches of particularly abundant resources that they recall visiting [46], or away from places where predators have been recently encountered [46].

Once this exploratory phase is over and the home range established, the animal's movement mechanisms may simply be described as a bias towards desirable sites. This idea naturally leads to the use of site fidelity models as good way to estimate home ranges from data, the so-called movement kernel density estimator (MKDE) [47]. By explicitly incorporating movement processes, such as Brownian bridges [9], these models can give better estimation of home range distributions than traditional methods such as (ordinary) kernel density estimation [8] or minimum convex polygons [6]. It remains an interesting open question as to whether MKDE can be improved further by the inclusion of territorial interactions.

## 8. ‘Non-mechanistic’ territory models

Over a decade ago, Adams [48] made a thorough review of territorial models, including mechanistic models. However, the term ‘mechanistic’ was used in a much broader sense than in this paper, and included ‘geometric models’ of territory borders, whereby the territory is assumed to exist *a priori*, but its size and shape are affected by the behaviour of its inhabitants. For example, Adams [49] describes a model of fire ants (*Solenopsis invicta*), where the pressure on a territory border increases with biomass and decreases with the square of the distance to the nest site. This is used to predict the relative sizes and shapes of neighbouring territories. However, the reasons behind the choice of these particular determinants of territory pressure are purely descriptive, and not derived from underlying processes. Models of the ants' movements and interactions such as reviewed in this paper could potentially help parametrize this model in a more mechanistic, and less speculative, way.

Adams [48] also reviews game-theoretic cost–benefit models and models of territory establishment. While the former have since been integrated into the mechanistic framework [42], the latter have yet to be understood from detailed descriptions of individual movements and interactions. The phenomenon of dispersal and re-establishment, often by adolescent animals, is very important for understanding population dynamics, disease spread and range expansion. Models that have been so far proposed in this regard tend to be based around the work of Fretwell & Lucas [50] which posits that an animal will establish a territory wheresoever its fitness is maximized, often using an economic cost–benefit framework [51]. Although some models have considered the costs of movement and interactions, e.g. Stamps & Krishnan [52], and more recent studies have modelled movement on a course scale of approximately 10 time steps per lifetime [53], to the best of our knowledge, none explicitly model the fine-scale movement and interactions that take place during territory establishment. Incorporating these ideas may give a more accurate understanding of the territorial dynamics that occur during dispersal and re-establishment.

## 9. Unsolved problems and future directions

Although mechanistic models have been successfully used to test hypotheses about the processes that cause territorial patterns to form, e.g. [13,14], the approach is typically based around testing which model fits the data best out of a set of hypothesized models, without seeking to understand how close the best model is to empirical reality. This is a major shortcoming for two reasons. First, though the best model may be significantly better than the others, this does not mean that it is sufficient to describe the data with enough accuracy to make accurate predictions about possible future scenarios. Second, without a quantitative measurement of closeness of a model to the data, it is not possible to tell when the model is complex enough to have identified all the key processes underlying territory formation. If mechanistic models are going to help turn ecology into a truly predictive science, then there is a pressing need to fill this gap.

Another challenge is to understand better how and when the IBM and advection–diffusion approaches differ, and when each should be applied. Typically, mean-field partial differential equation (PDE) approaches work well when there are large numbers of individuals. In other circumstances, as is often the case with territorial animals where a single individual or pack is defending the territory, it makes sense to check results of PDE studies against the underlying IBM to ensure that the predictions are accurate.

The main advantage of the PDE approach is that it gives analytic formulae that obviate the need for excessive simulation analysis. Thus, as long as the results are similar to the underlying IBM, such analysis is very convenient. While there exist accurate analytic approximations to the IBM territory models proposed so far, they do not explicitly incorporate the territorial interaction parameter, *T*_AS_ [18,33,34]. To remedy this, it is necessary either to create an analytic model that links the border movement to the interaction process, a programme that was initiated in [35], or to construct more accurate deterministic approximations than traditional mean-field methods allow. One possible avenue in the latter direction might be to use van Kampen's methods [54], which have successfully been used to find analytic reasons for disparities between mean-field and IBM approaches in biological systems [31].

All mechanistic models so far have been based around what might be called ‘stigmergent’ interactions [26]. That is, interactions that are mediated by modification of the environment. A classic example is scent or pheromone deposition. One animal deposits scent, adding to the environmental cues at that point. Sometime later, another animal responds to this cue by altering its behaviour. Other stigmergent processes include visual cues or vocal cues. The latter do not persist in the environment *per se* but rather exist in other animals' cognitive maps of the environment, who hear the cue and may respond several days later to the memory of it by avoiding the area from whence it came [55].

While most applications of mechanistic territory models so far have been regarding scent-marking mammals, it is straightforward to translate the ideas to other stigmergent processes, as evidenced by the use of this concept to model vocal cues in birds [20]. However, it is not so obvious how one might construct mechanistic models that incorporate direct interactions such as fighting and ritual displays, as observed in a variety of species [56]. In some bird populations, for example, neighbours may actively move every so often to a specific place on the territory border, whereupon they challenge the neighbouring flock to a territorial battle, which often consists of an aggressive display rather than actual physical contact. The outcome of such a battle may determine whether or not one of the flocks is able to advance its boundary and increase its territory [55]. Such complex behaviour is perhaps tricky to model and analyse from a mechanistic perspective, but is a necessary aspect to examine in order to understand fully how territories form and change.

We end by reiterating the idea that home range formation appears to be largely governed by one or both of two factors: territorial interactions and a cognitive map of the environment [57]. The latter may include various aspects of knowledge, such as those about resource availability, predation probability or other environmental covariates. One of the most important challenges for the future will be integrating these two important aspects of spatial localization to form an accurate, predictive theory of how space-use patterns emerge from the detailed, varied and complicated behaviours of interacting animals.

## References

[1] Olina GP 1622 Uccelliera, overo Discorso della Natura e Proprietà di Diversi Uccelli, e in particolare di que che Cantano, con il Modo di Prendergli, Conoscergli, Allivargli e Matenergli. Rome, Italy See: http://gdz.sub.uni-goettingen.de/dms/load/img/?PPN=PPN479740488&IDDOC=278056.

[2] Nice MM 1941 The role of territory in bird life. Am. Midl. Nat. 26, 441–487. (10.2307/2420732)

[3] Levin SA Segel LA 1985 Pattern generation in space and aspect. SIAM Rev. 27, 45–67. (10.1137/1027002)

[4] Lewis MA Murray JD 1993 Modelling territoriality and wolf–deer interactions. Nature 366, 738–740. (10.1038/366738a0)

[5] Shigesada N Kawasaki K Teramoto E 1979 Spatial segregation of interacting species. J. Theor. Biol. 79, 83–99. (10.1016/0022-5193(79)90258-3) 513804

[6] Harris S Cresswell WJ Forde PG Trewhella WJ Woollard T Wray S 1990 Home-range analysis using radio-tracking data: a review of problems and techniques particularly as applied to the study of mammals. Mammal Rev. 20, 97–123. (10.1111/j.1365-2907.1990.tb00106.x)

[7] Manly BF McDonald LL Thomas DL McDonald TL Erikson WP 2002 Resource selection by animals: statistical design and analysis for field studies, 2nd edn New York, NY: Chapman and Hall.

[8] Worton BJ 1989 Kernel methods for estimating the utilization distribution in home-range studies. Ecology 70, 164–168. (10.2307/1938423)

[9] Benhamou S 2011 Dynamic approach to space and habitat use based on biased random bridges. PLoS ONE 6, e14592 (10.1371/journal.pone.0014592) 21297869 PMC3027622

[10] Levin SA 2012 Towards the marriage of theory and data. Interface Focus 2, 141–143. (10.1098/rsfs.2012.0006) 23565329 PMC3293202

[11] Turchin P 1998 Quantitative analysis of movement: measuring and modeling population redistribution in animals and plants. Sunderland, MA: Sinauer Associates.

[12] Okubo A Levin SA 2002 Diffusion and ecological problems: modern perspectives, 2nd edn New York, NY: Springer.

[13] Moorcroft PR Lewis MA Crabtree RL 1999 Home range analysis using a mechanistic home range model. Ecology 80, 1656–1665. (10.1890/0012-9658(1999)080[1656:HRAUAM]2.0.CO;2)

[14] Moorcroft PR Lewis MA Crabtree RL 2006 Mechanistic home range models capture spatial patterns and dynamics of coyote territories in Yellowstone. Proc. R. Soc. B 273, 1651–1659. (10.1098/rspb.2005.3439) PMC1704082 16769637

[15] Moorcroft PR Lewis MA 2006 Mechanistic home range analysis. Princeton, NJ: Princeton University Press.

[16] Smith LM Bertozzi AL Brantingham PJ Tita GE Valasik M 2012 Adaption of an ecological territorial model to street gang spatial patterns in Los Angeles. Discrete Contin. Dyn. Syst. 32, 3223–3244. (10.3934/dcds.2012.32.3223)

[17] Giuggioli L Potts JR Harris S 2011 Animal interactions and the emergence of territoriality. PLoS Comput. Biol. 7, 1002008 (10.1371/journal.pcbi.1002008) PMC3053310 21423708

[18] Potts JR Harris S Giuggioli L 2012 Territorial dynamics and stable home range formation for central place foragers. PLoS ONE 7, e34033 (10.1371/journal.pone.0034033) 22479510 PMC3316599

[19] Potts JR Harris S Giuggioli L 2013 Quantifying behavioural changes in territorial animals caused by sudden population declines. Am. Nat. 182, E73–E82. (10.1086/671260) 23933730

[20] Potts JR Mokross K Lewis MA Submitted. A unifying framework for quantifying the nature of animal interactions. See http://arxiv.org/abs/1402.1802. 10.1098/rsif.2014.0333 PMC4032549 24829284

[21] Burt WH 1943 Territoriality and home range concepts as applied to mammals. J. Mammal 24, 346–352. (10.2307/1374834)

[22] Börger L Dalziel B Fryxell JM 2008 Are there general mechanisms of animal home range behavior? A review and prospects for future research. Ecol. Lett. 11, 637–650. (10.1111/j.1461-0248.2008.01182.x) 18400017

[23] Briscoe BK Lewis MA Parrish SE 2002 Home range formation in wolves due to scent marking. Bull. Math. Biol. 64, 261–284. (10.1006/bulm.2001.0273) 11926117

[24] Moorcroft PR 2012 Mechanistic approaches to understanding and predicting mammalian space use: recent advances, future directions. J. Mammal 93, 903916 (10.1644/11-MAMM-S-254.1)

[25] Potts JR Bastille-Rousseau G Murray DL Schaefer JA Lewis MA 2014 Predicting local and non-local effects of resources on animal space use using a mechanistic step-selection model. Methods Ecol. Evol. 5, 253–262. (10.1111/2041-210X.12150) 25834721 PMC4375923

[26] Giuggioli L Potts JR Rubenstein DI Levin SA 2013 Stigmergy, collective actions and animal social spacing. Proc. Natl Acad. Sci. USA 110, 16 904–16 909. (10.1073/pnas.1307071110) PMC3801015 24082100

[27] Fortin D Beyer HL Boyce MS Smith DW Duchesne T Mao JS 2005 Wolves influence elk movements: behavior shapes a trophic cascade in Yellowstone National Park. Ecology 86, 1320–1330. (10.1890/04-0953)

[28] Patterson TA Thomas L Wilcox C Ovaskainen O Matthiopoulos J 2008 State–space models of individual animal movement. Trends Ecol. Evol. 23, 87–94. (10.1016/j.tree.2007.10.009) 18191283

[29] Lewis MA White KAJ Moorcroft PR 1997 Analysis of a model for wolf territories. J. Math. Biol. 35, 749–774. (10.1007/s002850050075)

[30] Bateman AW Lewis MA Gall G Manser MB Clutton-Brock TH Submitted Territoriality and home-range dynamics in meerkats *Suricata suricatta* 10.1111/1365-2656.12267 24995457

[31] McKane AJ Newman TJ 2004 Stochastic models in population biology and their deterministic analogs. Phys. Rev. E 70, 041902 (10.1103/PhysRevE.70.041902) 15600430

[32] Huxley JS 1934 A natural experiment on the territorial instinct. Brit. Birds 27, 270–277.

[33] Giuggioli L Potts JR Harris S 2011 Brownian walkers within subdiffusing territorial boundaries. Phys. Rev. E 83, 061138 (10.1103/PhysRevE.83.061138) 21797333

[34] Giuggioli L Potts JR Harris S 2012 Predicting oscillatory dynamics in the movement of territorial animals. J. R. Soc. Interface 9, 1529–1543. (10.1098/rsif.2011.0797) 22262814 PMC3367816

[35] Potts JR Harris S Giuggioli L 2011 An anti-symmetric exclusion process for two particles on an infinite 1D lattice. J. Phys. A Math. Theor. 44, 485003 (10.1088/1751-8113/44/48/485003)

[36] Department for Environment, Food and Rural Affairs. 2011 The Government's policy on bovine TB and badger control in England See http://www.defra.gov.uk/.

[37] Krebs JR 1997 Bovine tuberculosis in cattle and badgers. London, UK: Ministry of Agriculture, Fisheries and Food (MAFF) Publications.

[38] Moorcroft PR Barnett A 2008 Mechanistic home range models and resource selection analysis: a reconciliation and unification. Ecology 89, 1112–1119. (10.1890/06-1985.1) 18481535

[39] Forester JD Im HK Rathouz PJ 2009 Accounting for animal movement in estimation of resource selection functions: sampling and data analysis. Ecology 90, 3554–3565. (10.1890/08-0874.1) 20120822

[40] Morrell LJ Kokko H 2005 Bridging the gap between mechanistic and adaptive explanations of territory formation. Behav. Ecol. Sociobiol. 57, 381–390. (10.1007/s00265-004-0859-5)

[41] Lewis MA Moorcroft PR 2001 ESS analysis of mechanistic home range models: the value of signals in spatial resource partitioning. J. Theor. Biol. 210, 449–461. (10.1006/jtbi.2001.2323) 11403565

[42] Hamelin FM Lewis MA 2010 A differential game theoretical analysis of mechanistic models for territoriality. J. Math. Biol. 61, 665–694. (10.1007/s00285-009-0316-1) 20033174

[43] Mahoney SP Virgl JA 2003 Habitat selection and demography of a nonmigratory woodland caribou population in Newfoundland. Can. J. Zool. 81, 321–334. (10.1139/z02-239)

[44] Grimm V Railsback SF 2005 Individual-based modeling and ecology. Princeton, NJ: Princeton University Press.

[45] Fagan WF 2013 Spatial memory and animal movement. Ecol. Lett. 16, 1316–1329. (10.1111/ele.12165) 23953128

[46] Avgar T Deardon R Fryxell JM 2013 An empirically parameterized individual based model of animal movement, perception, and memory. Ecol. Model. 251, 158–172. (10.1016/j.ecolmodel.2012.12.002)

[47] Benhamou S Riotte-Lambert L 2012 Beyond the utilization distribution: identifying home range areas that are intensively exploited or repeatedly visited. Ecol. Model. 227, 112–116. (10.1016/j.ecolmodel.2011.12.015)

[48] Adams ES 2001 Approaches to the study of territory size and shape. Annu. Rev. Ecol. Syst. 32, 277–303. (10.1146/annurev.ecolsys.32.081501.114034)

[49] Adams ES 1998 Territory size and shape in fire ants: a model based on neighborhood interactions. Ecology 79, 1125–1134. (10.1890/0012-9658(1998)079[1125:TSASIF]2.0.CO;2)

[50] Fretwell SD Lucas HL 1969 On territorial behavior and other factors influencing habitat distribution in birds. I. Theoretical development. Acta Biotheor. 19, 16–36. (10.1007/BF01601953)

[51] Loehle C 2013 Differential sorting of individuals in territorial species affects apparent habitat quality. J. Wildl. Manage. 77, 1166–1169. (10.1002/jwmg.574)

[52] Stamps JA Krishnan VV 1990 The effect of settlement tactics on territory sizes. Am. Nat. 135, 527–546. (10.1086/285060)

[53] Barraquand F Murrell DJ 2012 Evolutionarily stable consumer home range size in relation to resource demography and consumer spatial organization. Theor. Ecol. 5, 567–589. (10.1007/s12080-011-0148-7)

[54] van Kampen NG 1992 Stochastic processes in physics and chemistry. Amsterdam, The Netherlands: Elsevier.

[55] Jullien M Thiollay JM 1998 Multi-species territoriality and dynamic of neotropical understory bird flocks. J. Anim. Ecol. 67, 227–252. (10.1046/j.1365-2656.1998.00171.x)

[56] Maynard Smith J 1974 The theory of games and the evolution of animal conflicts. J. Theor. Biol. 47, 209–221. (10.1016/0022-5193(74)90110-6) 4459582

[57] Spencer WD 2012 Home ranges and the value of spatial information. J. Mammal 93, 929–947. (10.1644/12-MAMM-S-061.1)

